# Identification of metagenes and their Interactions through Large-scale Analysis of *Arabidopsis* Gene Expression Data

**DOI:** 10.1186/1471-2164-13-237

**Published:** 2012-06-13

**Authors:** Tyler J Wilson, Liming Lai, Yuguang Ban, Steven X Ge

**Affiliations:** 1 Department of Mathematics and Statistics, South Dakota State University, Box 2220, Brookings, SD, 57007, USA

## Abstract

**Background:**

Many plant genes have been identified through whole genome and deep transcriptome sequencing and other methods; yet our knowledge on the function of many of these genes remains limited. The integration and analysis of large gene-expression datasets gives researchers the ability to formalize hypotheses concerning the functionality and interaction between different groups of correlated genes.

**Results:**

We applied the non-negative matrix factorization (NMF) algorithm to the AtGenExpress dataset which consists of 783 microarray samples (29 separate experimental series) conducted on the model plant *Arabidopsis thaliana*. We identified 15 metagenes, which are groups of genes with correlated expression. Functional roles of these metagenes are established by observing the enriched gene ontology (GO) categories using gene set enrichment analyses (GSEA). Activity levels of these metagenes in various experimental conditions are also analyzed to associate metagenes with stimuli/conditions. A metagene correlation network, constructed based on the results of NMF analysis, revealed many new interactions between the metagenes. Comparison of these metagenes with an earlier large-scale clustering analysis indicates many statistically significant overlaps.

**Conclusions:**

This study identifies a network of correlated metagenes composed of *Arabidopsis* genes acting in a highly correlated fashion across a broad spectrum of experimental stimuli, which may shed some light on the function of many of the un-annotated genes.

## Background

### Previous Gene Co-expression Studies

In recent years, we have witnessed a deluge of new results coming from genome-wide microarray experiments, and the torrent of data seems likely to increase in the future. In particular, thousands of microarray data sets from experiments on the organism *Arabidopsis thaliana* based upon the Affymetrix ATH1 GeneChip microarray platform have been accumulating in data repositories such as GEO [1], ArrayExpress [2], and NASCArrays [3]. Gene co-expression analysis has emerged as a powerful tool in analyzing this information. In contrast to standard microarray experiments in which gene expression levels in a set of control and treated plants are measured and compared to find differentially expressed genes, co-expression analyses work on much larger datasets comprised of many experiments. Similar gene expression patterns can be observed across many treatments, instead of being limited to just one.

In general, gene co-expression studies fall into two broad categories: condition dependent and condition independent [4]. Condition independent studies use as many unique conditions as possible and a co-expression score between gene pairs (such as the Pearson correlation) is calculated. This analysis reveals underlying relationships between genes irrespective of tissue type, experimental stimuli, or developmental stage. Condition dependent studies are a more specific type of analysis, in which samples in the dataset are restricted by certain criteria such as tissue type or experimental stimuli such as abiotic stress or hormone treatment.

In an elegant condition dependent study by Bassel *et al.*[5], 138 microarray samples from imbibed mature *Arabidopsis* seeds were used to investigate genes involved with seed germination. Pearson correlations between gene expression profiles were calculated, and agglomerative hierarchical clustering revealed the existence of three large clusters of gene interactions. The gene correlation network created in the study revealed connections between genes known to be involved in the regulation of seed germination, with other genes whose function was unknown.

The idea of ‘guilt by association’, forming hypotheses concerning the biological role of genes based upon similar patterns of gene expression, plays an important role in co-expression analysis [4]. In a study by Lee *et al.*[6], a probabilistic network (AraNet) of functional associations was constructed using network-guided guilt by association. In this study, a diverse collection of functional genomics, proteomics, and comparative genomics data sets for *Arabidopsis* were integrated together. Functional gene relationships were then calculated based upon this extensive experimental data to form AraNet. The biological role of new candidate genes is inferred by measuring their network associations with genes in AraNet whose functional role has been determined experimentally. A similar approach was taken in a condition-dependent study by Schmid *et al*. [7] which analyzed global gene expression patterns from the AtGenExpress data series taken from samples covering the developmental stages of *Arabidopsis*. Results from this study indicate substantial overlap in gene expression activity between samples. Interestingly, histograms of relative gene expression values taken from samples from the root, leaf, apex, and pollen samples of the plant showed marked differences.

In a condition independent study by Atias *et al.*[8], gene expression profiles from 43 microarray experiments conducted on *Arabidopsis thaliana* were analyzed. Their data is comprised of 857 hybridization samples collected under a wide variety of experimental conditions at different times and across 37 different laboratories. Within the microarray samples comprising each experiment in the experimental series, they calculated the Pearson correlation coefficient between all gene pairs. Only highly correlated gene pairs appearing simultaneously in 20 of the 43 experiments were considered for further analysis. A novel scoring function developed for the study allowed them to measure the degree of correlation for gene expression profiles spanning the entire dataset. This was then used to develop a gene correlation network. An excellent discussion and overview of different co-expression studies is presented in a study by Usadel *et al*. [4].

### The Model Organism *Arabidopsis thaliana*

*Arabidopsis thaliana* is popular as a model organism in the plant sciences. It has one of the smallest genomes in the plant kingdom (125 Mbp), approximately 28,000 genes. Currently the function of about half of these genes are unknown. *Arabidopsis* fulfils the same role in plant biology that *Drosophila melanogaster* and mice play in animal science. Although *Arabidopsis* is not an agriculturally useful plant, many of the genes within its genome are homologous to genes in plants with more complex genomes, and often play similar roles within these organisms. It is particularly useful in studying the biology of flowering plants. This is the primary reason we chose to focus on *Arabidopsis* in our research. By formulating biological hypotheses concerning many of the unknown genes, we hope our research will serve as a guide for planning future microarray experiments.

### The NMF Algorithm

The NMF algorithm was first introduced in 1999 by Lee and Seung [9]. It is in the same class as other dimensionality-reducing algorithms like Principal Component Analysis (PCA) and Vector Quantization (VQ). In contrast to the more holistic data representations returned by PCA and VQ methods, the NMF algorithm provides a local, parts-based representation of the data. When applied to a gene expression dataset, the NMF algorithm finds metagenes composed of correlated genes representing a local or global gene expression pattern. In contrast to more traditional clustering methods such as hierarchical clustering, genes can appear in multiple metagenes and are not constrained by the algorithm to be a member of only one cluster. The NMF algorithm is capable of finding clusters of genes co-expressed on a small set of experiments as well as genes co-expressed globally. More importantly, the algorithm allows genes to be in different clusters at different activation levels, instead of being placed in just one cluster. Both of these features make the NMF method an ideal candidate for analyzing the AtGenExpress dataset.

NMF has been applied with considerable success to gene expression datasets other than *Arabidopsis*[10-16]. In a study by Brunet *et al.*[17] the NMF algorithm was used to reveal cancer subtypes by clustering human tumor samples, and to find metagenes involved in leukemia and brain cancer datasets. They also developed a novel method for finding the optimum number of metagenes intrinsic to a dataset. We incorporated this method into our study.

## Results

### The AtGenExpress Dataset

AtGenExpress is a large global research project whose purpose is to discover the transcriptome of the model organism *Arabidopsis thaliana*. The datasets were downloaded from the NCBI Gene Expression Omnibus [1]. The combined dataset is comprised of 783 samples over 29 different experimental series (see Table 1). This is a condition independent study involving many different type of stimuli and experimental conditions. Since NMF algorithm has been applied with much success in gene co-expression studies on human tissue samples, we were interested to see how it would fare when applied to *Arabidopsis*. We are looking for genes co-expressed locally on specific experiments as well as globally.

**Table 1:** AtGenExpress Experimental Series

**GSE Accession Number**	**Number of Samples**	**Experiment Description**	**Sampled Tissue**
GSE5615	42	Response to bacterial-(LPS, HrpZ, Flg22) and oomycete-(NPP1) derived elicitors	Leaf
GSE5616	18	Response to Phytophthorainfestans	Leaf
GSE5617	48	Light treatments	Shoot
GSE5620	36	Stress Treatments (Control plants)	Root and shoot
GSE5621	24	Stress Treatments (Cold stress)	Root and shoot
GSE5622	24	Stress Treatments (Osmotic stress)	Root and shoot
GSE5623	24	Stress Treatments (Salt stress)	Root and shoot
GSE5624	28	Stress Treatments (Drought stress)	Root and shoot
GSE5625	24	Stress Treatments (Genotoxic stress)	Root and shoot
GSE5626	28	Stress Treatments (UV-B stress)	Root and shoot
GSE5627	28	Stress Treatments (Wounding stress)	Root and shoot
GSE5628	32	Stress Treatments (Heat stress)	Root and shoot
GSE5629	24	Developmental series (seedlings and whole plants)	Shoot and whole plant
GSE5630	60	Developmental series (leaves)	Leaf (different stages)
GSE5631	21	Developmental series (roots)	Root (different stages)
GSE5632	66	Developmental series (flowers and pollen)	Flower (different stages)
GSE5633	42	Developmental series (shoots and stems)	Shoots and stems (different stages)
GSE5634	24	Developmental series (siliques and seeds)	Siliques and seeds (different stages)
GSE5684	12	Pathogen Series: Response to Botrytis cinerea infection	Mature leaf
GSE5685	32	Pathogen Series: Pseudomonas half leaf injection	Stage 10–11 rosette leaf
GSE5686	48	Pathogen Series: Response to Erysipheorontii infection	Mature leaf
GSE5687	4	Different temperature treatment of seeds	Seed
GSE5688	22	Response to sulfate limitation	Root
GSE5696	26	Effect of brassinosteroids in seedlings	Whole plant
GSE5697	8	Comparison of plant hormone-related mutants	Whole plant
GSE5698	12	Cytokinin treatment of seedlings	Whole plant
GSE5699	6	ARR21C overexpression	Whole plant
GSE700	8	Effect of ABA during seed imbibition	Seed
GSE701	12	Basic hormone treatment of seeds	Seed

### Selecting the Dimensionality Reduction Parameter for the NMF Algorithm

**Figure 1:**
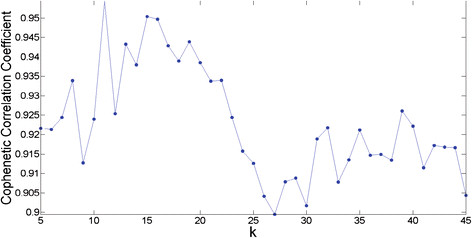
**Cophenetic Correlation Coefficient for the determination of optimal number of metagenes *k.*** Peaks in this plot represent stable *k* values, but we were also looking for consistency. The value *k* = 15 was chosen for this study because it is a high peak with a slow drop-off.

An important part of the NMF algorithm is that it reduces the dimensionality of the original data space to a much smaller dimension *k*. Thus it is important that we choose the optimal value for *k* which decomposes the dataset into *k* metagenes/encoding coefficients. In Figure 1, the Cophenetic correlation coefficient (CCC) is plotted against different *k* values in the range *k* = 5…45. The CCC is a measure which quantifies the stability of a dimensionality reduction parameter *k*. For stable values, it will be close to 1, and for unstable values, it will be close to 0. Peaks in the plot represent stable values, but we were also looking for consistency (a peak followed by a slow drop-off). Based upon these considerations, we felt *k* = 15 was an optimal choice. The method for generating this graph was adopted from the study by Brunet *et al.*[17] and is explained in the Methods section.

### The Metagenes and Encoding Coefficients

Analysis of the dataset involved applying the NMF algorithm to reduce the dimensionality of the data to a set of metagenes, and associated encoding coefficients. Each metagene represents a collection of genes behaving in a functionally correlated fashion within the genome. The encoding coefficients express the degree of activation of each metagene on each sample within the dataset. See the Methods section for details.

Each gene within a metagene has an activation level which represents the degree to which that gene is expressed. The metagenes were sorted in descending order according to the activation levels of their genes, and the genes within each metagene were also sorted in descending order. Metagene 1 represents the most highly expressed metagene within the dataset, and Metagene 15 the least highly expressed. Some of the genes in the metagenes have been well-studied, while others have not been annotated at all. Table 2 below displays the top 20 genes involved in metagene 1. A list of all the metagenes is given in Additional file 1.

**Table 2:** The top 20 Most Signficant Genes for Metagene 1

**TAIR ID**	**GenBank ID**	**Description**
At1g80840	BAA87058	Transcription metagene, putative similar to WRKY transcription metagene
At1g05575	---	Expressed protein
At1g19180	---	Unknown protein
At4g29780		Hypothetical protein
At1g27730	CAA64820	Salt-tolerance zinc finger protein identical to salt-tolerance zinc finger
At4g34410	---	Putative protein ethylene-responsive element binding protein homolog, Stylosantheshamata, U91857
At1g76650	CAA56517	Putative calmodulin similar to calmodulin
At2g34600	---	Hypothetical protein predicted by genscan
At2g26530	D88743	AR781, similar to yeast pheromone receptor
At1g19020	---	Expressed protein ; supported by full-length cDNA: Ceres: 31015
At4g24570	---	Putative mitochondrial uncoupling protein mitochondrial uncoupling protein
At4g17490	---	Ethylene responsive element binding metagene-like protein (AtERF6)
At3g55980	---	Putative protein zinc finger transcription metagene (PEI1)
At3g01830	CAB42906	Hypothetical protein similar to calmodulin-like protein
At5g42380	---	Putative protein contains similarity to calmodulin
At1g72520	CAB56692	Putative lipoxygenase similar to lipoxygenase
At2g32210	---	Unknown protein
At3g25780	---	Unknown protein ; supported by full-length cDNA: Ceres:3457
At1g61340	---	Late embryogenesis abundant protein, putative similar to late embryogenesis abundant protein
At4g30280	---	xyloglucan endo-1,4-beta-D-glucanase-like protein

### Metagene Involvement in Experimental Series

**Figure 2:**
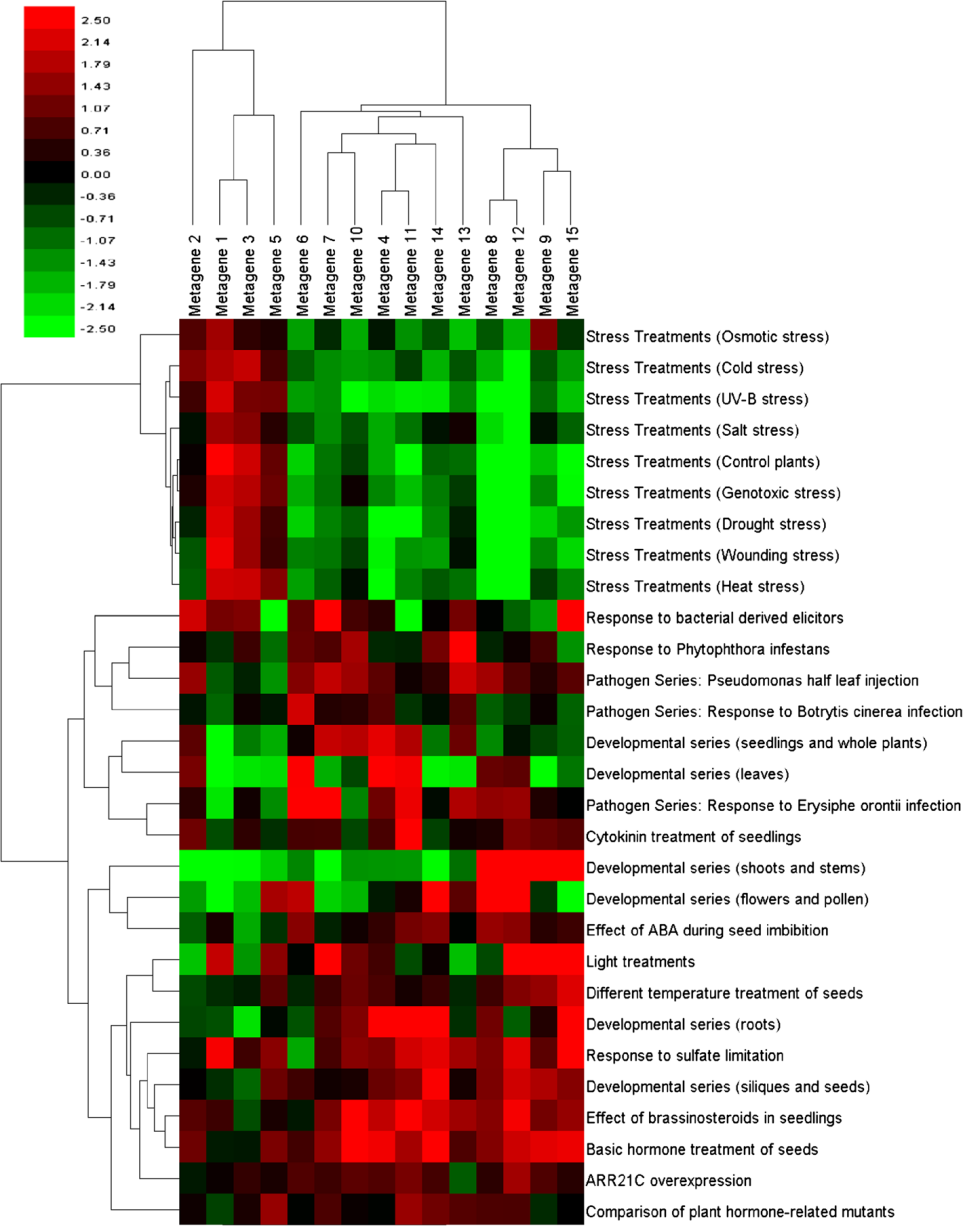
**Metagene Activity in Experimental Series.** This heat map shows the *z*-values for all metagenes for each experimental series in the dataset. Red indicates a metagene is more active in an experimental series, and green indicates it is suppressed.

The encoding coefficients returned by the NMF algorithm measure the degree to which each metagene is active in each of the 783 samples within the dataset. Multiple samples comprise an experiment, and through an application of the Kruskal-Wallis test [18], it is possible to determine on which series of experiments each metagene is active on. Figure 2 is a heat map of the *z*-values returned by the Kruskal-Wallis test for each experiment in the AtGenExpress dataset. Information about how this figure was generated can be found in the Methods section.

One striking feature in Figure 2 is that metagenes 1, 3 and 5 are the dominant metagenes involved in the stress series of experiments, and appear to suppress the other metagenes in that series. These metagenes also represent the most actively expressed metagenes our analysis uncovered. Results from examining their functional annotation in DAVID (http://david.abcc.ncifcrf.gov/) [19,20] appear to confirm this. The top five most highly enriched categories for the three metagenes are shown in Table 3. Metagenes 1 and 5 are primarily involved in responses to stimuli (chemical or otherwise). Metagene 3 is involved in photosynthetic responses related to light stimuli, as well as the generation of precursor metabolites used in catabolic and anabolic pathways to generate ATP or in the synthesis of more complicated organic chemicals such as lipids, amino acids, and nucleotides. Another interesting feature of Figure 2 is that metagenes 8, 12, 9 and 15 appear very active in the Developmental series related to shoots and stems, while all of the other metagenes are suppressed.

**Table 3:** Top 5 Most Enriched GO-BP categories for Metagenes 1, 3, 5

**Biological Process**	**P-Value**	**FDR**	**Metagene**
Response to chitin	4.08E-13	5.13E-10	1
Response to chemical stimulus	2.82E-12	3.55E-09	1
Response to stimulus	2.39E-11	3.00E-08	1
Response to carbohydrate stimulus	2.40E-11	3.02E-08	1
Response to stress	8.84E-11	1.11E-07	1
Photosynthesis	1.71E-74	2.62E-71	3
Photosynthesis, light reaction	2.53E-39	3.88E-36	3
Generation of precursor metabolites	3.01E-31	4.60E-28	3
Photosynthesis, light harvesting	1.49E-20	2.29E-17	3
Photosynthetic electron transport in photosystem I	1.49E-16	1.67E-13	3
Response to stimulus	1.83E-12	2.78E-09	5
Response to stress	9.95E-09	1.52E-05	5
Response to chemical stimulus	2.74E-08	4.17E-05	5
Photosynthesis	7.35E-07	1.12E-03	5
Response to abiotic stimulus	9.79E-07	1.49E-03	5

### Functional Characterization of Metagenes Using Gene Set Enrichment Analysis

GSEA [21] was applied to the metagenes returned by NMF. The GSEA algorithm accepts as one of its inputs a ranked list of genes. The ranking scheme is specified by the researcher to highlight some feature they are interested in. Since the coefficient of a gene in a metagene correlates to a general degree of expression within that metagene (the higher the coefficient value, the more active the gene), the metagene coefficients provided a useful way to rank the genes in the metagenes for input into GSEA.

**Figure 3:**
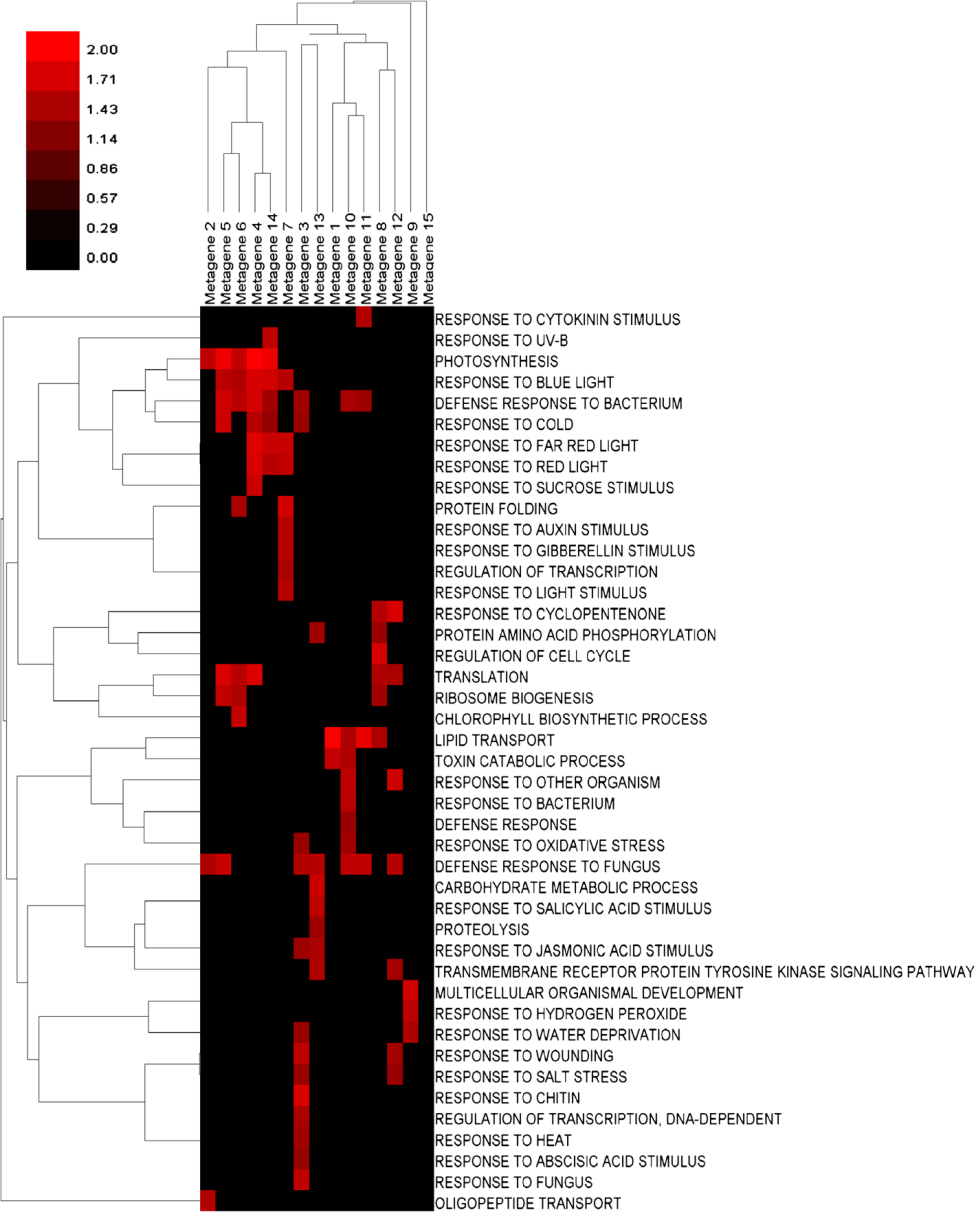
**Metagene GSEA Enrichment in Biological Processes.** The NES score plotted in this heat map is a measure of metagene enrichment within a specific gene ontology involved with biological processes. Bright red cells indicate high enrichment.

Metagene involvement in three gene ontologies (molecular functions, cellular components, and biological processes) was examined and the results for the biological processes ontology are summarized in Figure 3. Only gene ontologies enriched with a nominal p-value of less than 0.01 were examined.

The NES (Nominal Enrichment Score) is a measure of the enrichment of a metagene within a gene ontology – and is the value plotted in Figure 3. The interpretation of this measurement is discussed in the Methods section on GSEA, and the spreadsheets from which this heat map was generated (as well as the other two gene ontologies investigated) are included in Additional file 2, 3, and 4. Results for the cellular components and molecular functions ontology are summarized in Additional file 5: Figure S9 and S10, respectively.

In Figure 3, some metagenes appear to be involved in a number of different biological processes, while others are involved in only a few. Metagene 3 is active on biological process ontologies involving responses to light, as well as those involved with defense responses to bacterium and funguses. It is also active in a variety ontologies related to stress response experiments such as water deprivation and response to wounding. An examination of Figure 2 reveals that it is a dominant metagene involved in the stress series of experiments. In contrast to this, metagene 2 is involved only in three different biological process ontologies (photosynthesis, defense response to fungus, and oligopeptide transport). Looking at Figure 2 reveals that it is strongly suppressed on the developmental series of experiments related to shoots and stems, but expressed on experiments for responses to bacterial derived elicitors. It is not very active on the other experimental series.

### Metagenes 1, 3 and 5

We observed in Figure 2 that metagenes 1, 3, 5 were the principal metagenes involved in the stress series of experiments. A closer look at the GSEA analysis for these metagenes appears to confirm this.

**Figure 4:**
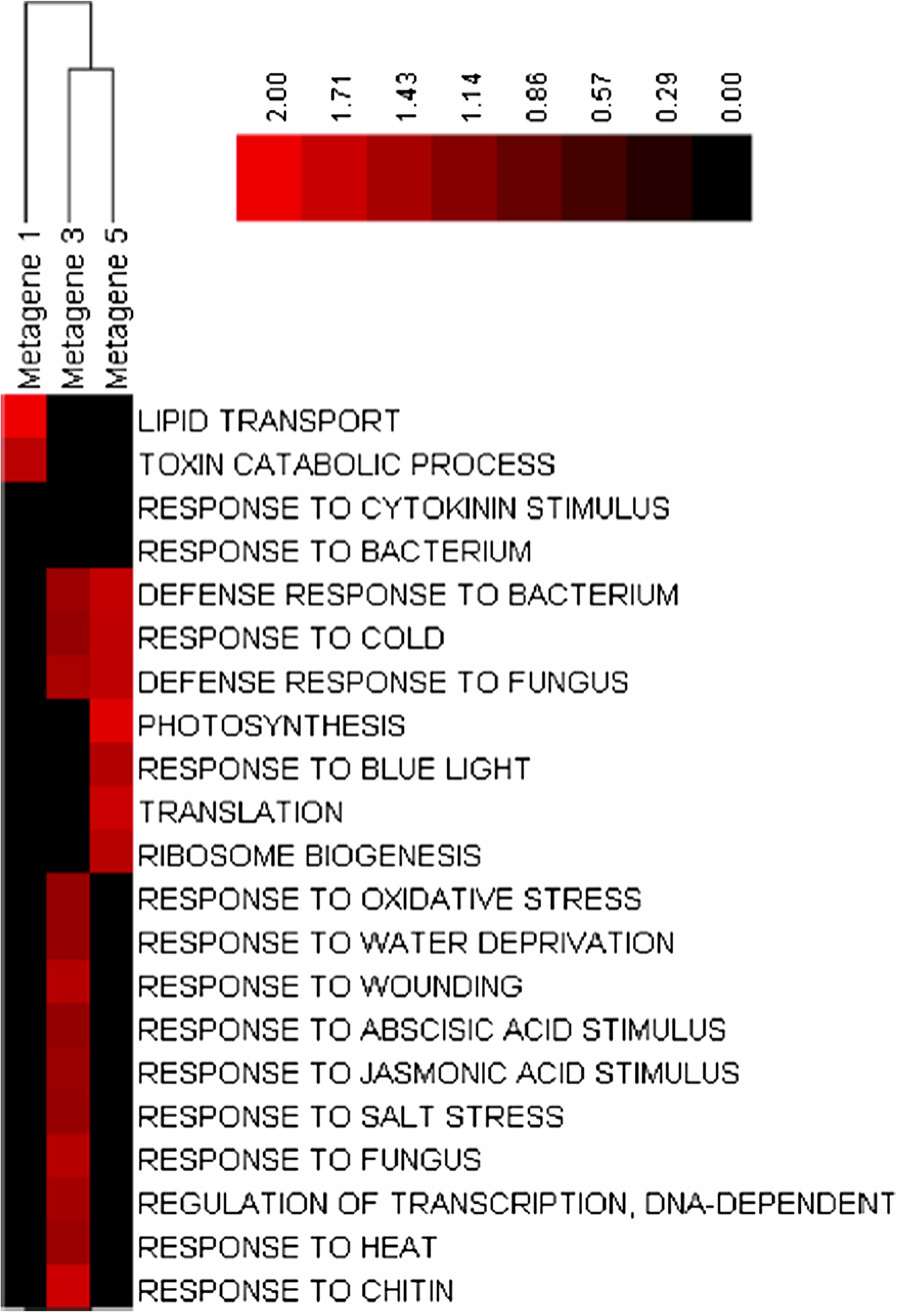
**Metagene 1,3 and 5 Activity in Biological Processes.** This heat map shows the biological process NES score for metagenes 1, 3 and 5. Bright red cells indicate high enrichment within an ontology. Metagene 3 is highly enriched with respect to different responses related to chemical and mechanical stimulus. Metagene 5 is enriched with respect to defense responses such as light and bacterial infection. Metagene 1 is also enriched in catabolic processes related to toxin removal, which one would expect for a metagene active under the stress series of experiments.

In Figure 4, metagene 3 is enriched in a number of defense responses and responses to a wide variety of stimuli. Moreover, metagene 5 is involved in a number of defense responses and responses to stimuli, and is also enriched in categories such as ribosome biogenesis and photosynthesis. Metagene 1 is heavily enriched in lipid transport and catabolic processes involved in the breakdown of toxins – which would certainly make sense if it was active in the stress series of experiments.

### Metagene Correlation Network

To determine how the metagenes interact with each other, a Spearman correlation matrix *C* was calculated by computing all pair-wise correlations between the metagenes.

1. Each network node represents a metagene.

2. The size of each node is proportional to the metagene activity within the dataset.

3. A line is drawn between a pair of nodes if the p-value of their Spearman correlation is less than 10^-12^.

a. Positive correlations are denoted by red lines

b. Negative correlations are denoted by green lines

c. The width of the line is proportional to the strength of the correlation.

4. The pie slices within each node represent the amount of enrichment for specific gene ontologies as determined by GSEA.

**Figure 5:**
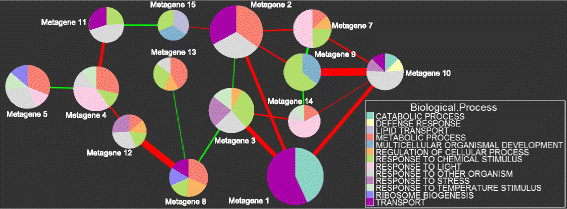
**Metagene Correlation Network for Biological Processes.** Each node in this network represents a metagene. The size of each node is proportional to the activity of the metagene within the dataset. The width of lines between a pair of nodes is proportional to the strength of the correlation between them. Positive correlations are denoted by red lines, and negative correlations by green lines. Only Spearman correlations with a p-value less than 10^-12^ are visible. The pie slices within each node represent the amount of enrichment for specific gene ontologies (the NES score).

In the correlation network of Figure 5, metagene 1 is comprised of genes which are the most actively expressed across all samples within the dataset. It has a very strong positive correlation with metagenes 3 and 10. Metagenes 9 and 10 have a strong positive correlation. A close examination of the Kruskal-Wallis *z*-values for these two metagenes confirms this. Metagene 9 is strongly expressed when metagene 10 is the most active during the stress series of experiments, and both metagenes are suppressed during the pathogen and developmental series of experiments. Correlation networks for the gene ontologies of cellular components and molecular functions are available in Additional file 5: Figure S5 and Figure S6, respectively.

### A Comparison of Metagenes with Atias *et al.*

In the study by Atias *et al*. [8] a scoring function was developed that measures the correlation between gene pairs across different experimental series. The methodology for selecting and clustering genes in the Atias study is fundamentally different than those employed here, so comparing the results from Atias with ours is useful to verifying the results of this study.

Atias created three different gene correlation networks based on the scoring threshold. For the 0.3-threshold network, gene pairs with a score less than 0.3 were filtered out of the analysis. Using graph theoretic methods encapsulated within the MCODE plug-in for Cytoscape [22], 38 clusters of genes were identified. The same process was repeated to create the 0.4-threshold network (which was smaller), and identified 35 clusters. A network generated from the pathogen series of experiments within their dataset was also created. This data subset includes microarray samples from 8 of the 43 *Arabidopsis* experiments involved with pathogen treatments. Within this network, MCODE identified 15 gene clusters.

**Figure 6:**
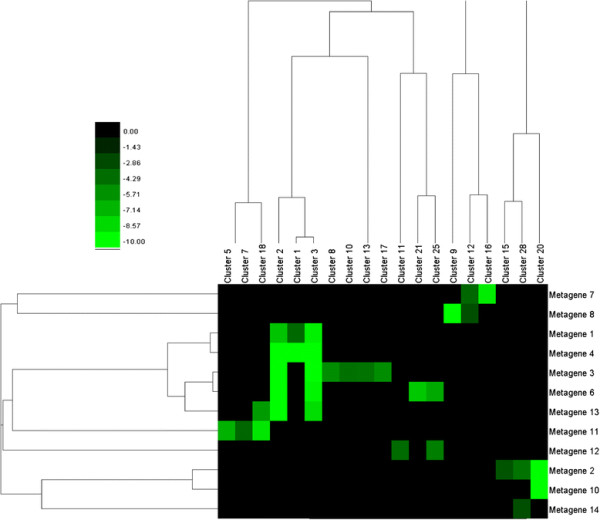
**Heat map of intersection p-values for the 0.3-threshold network.** The intersection between clusters in the Atias study for the 0.3 threshold-network, and the metagenes in this study are visualized as a heat map, with cells representing log-10 transformed p-values from a hypergeometric test. Bright green values indicate significant statistical overlap (very low p-values).

**Figure 7:**
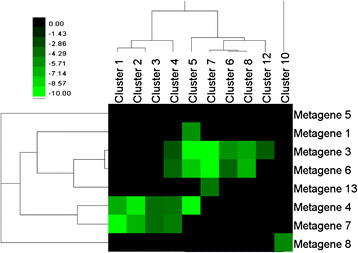
**Heat map of intersection p-values for the pathogen network.** Intersection p-values between clusters in the pathogen network from Atias, and the metagenes in this study. Bright green cells represent significant statistical overlap. The intensity of the cells represents a log-10-transformed p-value returned by the hypergeometric test.

A hypergeometric test was used to determine the degree of intersection between the metagenes in this study, and the clusters identified in the 0.3-threshold and pathogen networks of Atias. See the Methods section for details. A matrix of p-values for each of the networks was calculated. Hierarchical clustering analysis on both metagenes/clusters from Atias was then performed. The p-values in the heat maps shown in Figures 6–7 were log_10_ transformed, and the bright green squares correspond to p-values less than or equal to 10^-10^.

The Atias network with a score threshold of 0.3 contains 1372 genes. Of these, 1118 genes are also contained within the metagenes discovered in this analysis. Significant statistical overlaps were observed (see Figure 6) between metagenes 1 and 4, and clusters 1–3. Overlaps also occur between metagenes 2 and 10, and cluster 20. Metagene 3 exhibits strong overlaps between clusters 2, 3, 8, 10, 13, and 17 (the most of any metagene in our group). Most significantly (for the purpose of validating this study), metagenes 1–4 (the most significant metagenes identified in our analysis), exhibited very strong overlaps with a large number of clusters identified by Atias.

In both the study by Atias and our study, portions of each dataset are comprised of microarray samples involved in pathogen experiments. In this study, metagenes 6, 7, 8, and 13 are actively expressed in at least two out of the three pathogen series (with metagene 6 being involved in all three). See Additional file 5: Figure S7 for a heat map showing metagene involvement in the pathogen series.

Examining the p-values of intersection for these metagenes with the clusters from the pathogen network in the Atias study reveals many significant overlaps. Not surprisingly, metagene 6 intersects with the most clusters. See Additional file 5: Figure S8.

## Discussion

The three primary steps of the analysis conducted in this paper are:

1. Filtering genes of interest based upon their expression activity on the samples.

2. Finding the optimal number of metagenes intrinsic to the dataset and applying NMF. Using the results of NMF to construct a metagene correlation network.

3. Determining the functionality of the metagenes by applying GSEA, and using NMF results to provide a ranking for the genes within each metagene.

In the study by Lee and colleagues [23], as well as the study by Atias *et al*. [8], step 1 was accomplished by ranking gene-pairs based on their frequency of correlation within experiments in the dataset. The benefit of using this approach is that differences in scale/resolution between different experiments are no longer an issue because the Pearson correlation between two variables is invariant (up to a sign) to changes in location and scale of each variable. A drawback to this approach (and the reason we did not use it in this study) is that it preferentially selects genes which are highly correlated with other genes across a large number of samples. Genes which have correlated expression patterns with other genes on a small number of experiments could potentially be filtered out. The NMF algorithm is adept at finding genes like this, and so we chose a filtering scheme based on expression activity across all of the samples. Despite using a different methodology for gene selection, significant overlaps between genes in the Atias study and this one were observed.

A benefit of using the NMF algorithm instead of other algorithms such as hierarchical clustering, PCA, or VQ methods is that it does not constrain genes to belong exclusively to one cluster, so the method realistically models the way in which many genes perform different functional roles within the genome. There is also a way to approximate an optimum number of metagenes (see [17] and the Methods section), which is not true of many other clustering methods.

In Figure 1 in which the Cophenetic correlation coefficient is plotted for different values of the *k* parameter, more than one peak was observed (representing a stable clustering). A promising area of future research could be to calculate metagenes for different stable *k*-values and examine differences between them. It would also be interesting to see if applying GSEA analysis to metagenes resulting from higher *k*-values results in more functionally specific metagenes than smaller *k*-values.

## Conclusions

NMF analysis of the AtGenExpress dataset revealed 15 metagenes representing collections of genes with correlated expression patterns (both globally with respect to all of the experiments in the data series, and locally with respect to a smaller subset of the experiments). By combining the NMF results with *z*-values returned by the Kruskal-Wallis test, interesting interactions between the metagenes were revealed. Some of the metagenes appear to act in concert to suppress other metagenes for some of the experiments while they are active (such as metagenes 1, 3, 5 over the stress series of experiments). A metagene correlation network also revealed similar trends. Application of GSEA showed many interesting specializations of functions among the different metagenes, and it is hoped that the analysis presented here provides useful insight for planning future experiments.

## Methods

### Pre-processing

The AtGenExpress data files were downloaded from the NCBI Gene Expression Omnibus (http://www.ncbi.nlm.nih.gov/gds) [1]. The microarray platform the sample data comes from is the AffymetrixGeneChip *Arabidopsis* ATH1 Genome array. Each sample file contains gene expression information for 22,811 genes. The Robust Multi-array Average (RMA) algorithm provided by the Bioconductor package [24] available for R [25] was used to pre-process the CEL files from the dataset. RMA expression values for each of the 29 experiments in the data series were then combined into one data matrix of dimension *N*×*P* where *N* = 22,811 is the number of genes on the ATH1 array, and *P* = 783 is the number of samples.

### Filtering

Filtering of the dataset was conducted in two stages. For the first stage, the Wilcoxon sign-based present/absence detection algorithm available in Bioconductor [24] was applied to the data matrix. This is a statistical test that determines whether a gene expression signal is absent, present, or marginal. Genes for which 80 % or more of their expression values were determined to be absent or marginal were filtered from the dataset. The mean for each gene across all sample expressions was then subtracted row-wise to account for differences in magnitude of expression value due to probe sensitivity and mRNA levels.

The second level of filtering was applied by ranking all of the genes using the *L*_2_ norm. Let $g_{i}$, i=1…P , where $P=\left\{\text{number}\;\text{of}\;\text{samples}\right\}$, represent the RMA expression value of gene *g* for Sample *i*. The *L*_2_ norm of *g* is defined to be:

$${\left\|g\right\|}_{2}=\sqrt{{\left(g_{1}\right)}^{2}+{\left(g_{2}\right)}^{2}+\cdots +{\left(g_{p}\right)}^{2}}$$

This measure assigns a value to each gene based upon its overall degree of gene expression with respect to all of the samples in the dataset. The top 7000 genes ranked according to their *L*_2_ norm values were selected for further analysis.

### Adjusting for Batch Effects

After normalizing the dataset, it was necessary to adjust for the problem of batch effects. An Empirical Bayes (EB) method [26] was used to correct batch effects between samples. The EB method corrects for batch effects by making estimates of batch effect noise based on information gathered across all genes and experimental conditions within the data. It is also robust to outliers within batches of small sizes. The dataset for this study contains some experiments with a small number of samples, so this was an important factor in choosing this method.

To adjust for batch effects, the batches have to be first defined. Samples were grouped into the same batch if they were from the same experiment – resulting in 29 different batches.

The influence of batch effects on the data can be inferred by selecting two rows of the dataset at random. Each row represents a gene and its vector of expression values across all samples in the dataset. In the absence of batch effects, it is expected that the Pearson correlation coefficient between two randomly selected genes will be very close to zero.

To assess the effectiveness of the Empirical Bayes method, 1000 genes (rows) were selected at random from the dataset, and a plot of the standard deviation versus the average Pearson correlation between each gene and every other gene in the random sample was calculated. The plot of this graph is available as Additional file 5: Figure S1.

### Non-negative Matrix Factorization

After standard normalization, adjusting for batch effects, and filtering, the dataset consists of gene expression values for 7000 genes in 783 samples. It is represented by a matrix V of dimension N x P, where *N* = {number of genes}, *P* = {number of samples}.

Non-negative matrix factorization factors *V* into the product of two matrices *W* and *H*, where *W* has dimension *N*×*k*, and *H* has dimension *k*×*P*:

(1) V≈WH

*k* is chosen so that *k* is much smaller than *N*. The NMF factorization imposes a non-negativity constraint on *W* and *H*, so entries in both matrices are greater than 0. The method begins by randomly initializing *W* and *H*. It then iteratively updates the matrices to minimize a cost function (usually related to the measure: $\left\|V-WH\right\|$). At each iteration, *W* and *H* are updated by multiplying them by some factor which improves the quality of the approximation in Equation 1.

In this study, the *alternate non-negative least squares using projected gradient* NMF method first proposed by Lin [27] was chosen because of its speed and robustness. The algorithm was implemented in Matlab using the NMF: DTU toolbox available at: http://cogsys.imm.dtu.dk/toolbox/nmf/index.html[28].

### Finding the Optimal Number of Metagenes

Although the factor *k* which controls the number of metagenes in Equation 1 is specified by the user, there is a way to determine an optimal value of *k* which is determined by the structure of the dataset itself [17]. The matrix *H* in Equation 1 was used to group the *P* samples into *k* different clusters. Explicitly, if *h*_*ij*_ is the largest value in column *j* of *H*, then sample *j* was placed in cluster *i*.

The NMF algorithm does not always converge to the same solution each time it is run. However, if we have chosen a stable value for *k*, then the assignment of *P* samples into *k* clusters should not change significantly from one run to the next.

For each run, the sample clustering can be represented by a connectivity matrix *C* of dimension *P×P*. Set $c_{\mathrm{ij}}=1$ if samples *i* and *j* belong to the same cluster, and $c_{\mathrm{ij}}=0$ if they belong to different clusters. The consensus matrix $\bar{C}$ is defined as the average connectivity matrix after many runs. The entries $\bar{c_{\mathrm{ij}}}$ of $\bar{C}$ express the probability that samples *i* and *j* cluster together after many runs. For a clustering that is stable, we expect that most entries in $\bar{C}$ would be distributed near 0 or 1. The dispersion of entries in $\bar{C}$ serves as a metric which can be used to assess the stability of our choice of clustering parameter *k*. The heat maps of consensus matrices for different choices of *k* are displayed in Additional file 5: Figures S2-S3.

The stability of a clustering for a given value of *k* is quantified using the Cophenetic correlation coefficient (CCC) of the consensus matrix: $\rho_{k}\left(\bar{C}\right)$. *ρ*_*k*_ is calculated by computing the Pearson correlation coefficient between two distance matrices. The first distance matrix represents the distance between samples of $\bar{C}$ after applying hierarchical clustering. The second distance matrix is $I-\bar{C}$ and represents the distance between samples in the original consensus matrix. In a perfect clustering, all entries would be equal to 0 or 1 in the consensus matrix and $\rho_{k}\left(\bar{C}\right)=1$. In a more realistic consensus matrix, the entries are dispersed between 0 and 1, and $\rho_{k}\left(\bar{C}\right)<1$.

Consensus matrices were computed for *k* = 5…45. For each value of *k*, the NMF algorithm was run 50 times and the consensus matrix was computed by taking the average of these runs. The CCC was calculated for each value of *k*, and a plot of the CCC versus *k* values generated (see Figure 1). Peaks in the graph represent stable choices for *k*, but we were also looking for consistency (a peak with a slow drop-off). Based upon these considerations, *k* = 15 seemed like an optimum choice.

### The Metagenes

Each column of *W* in (1) represents a metagene. A metagene is a set of genes behaving in a functionally correlated manner within the genome. Entry *w*_*ij*_ represents the coefficient of gene *i* in metagene *j*. Genes which are more active in the genome have higher coefficient values. The expression pattern of genes in *V* are approximated by a linear combination of the *k* metagenes in *W*.

To compare the activity of the metagenes, the column sums of the coefficient values were calculated, and the columns sorted in descending order according to their column sums. So the first column of *W* represents the most active metagene, and the last column the least active.

A cut-off threshold of *δ* = 0.2 was set, so that gene coefficients with a value less than this were filtered out. Genes with coefficients greater than *δ* were included in the metagenes. Additional file 1 is a spreadsheet containing the list of metagenes and their genes, and Additional file 5: Figure S4 displays a histogram of the coefficient values for the metagenes, showing where the cut-off was made.

### Comparison with the result of Atias *et al*

The hypergeometric p-value returned by testing for statistical overlap between a gene cluster in the Atias study and a metagene in this one was calculated using Equation 2 below:

(2) $$p\mathrm{value}=\sum_{i=x}^{cs}\frac{\left({}_{\;i}^{\mathrm{ms}}\right)\left({}_{cs-i}^{N-ms}\right)}{\left({}_{\mathrm{cs}}^{N}\right)}$$

where:

*N* = 7000 (the number of genes in our filtered dataset)

*ms* = number of genes in the metagene

*cs* = number of genes in cluster from Atias study

*x* = number of genes in metagene which match genes in the Atias cluster

All calculations were carried out in R [25].

### Creating the Metagene Correlation Network

To determine how the metagenes interact with each other, a correlation matrix *C* was calculated by computing all pair-wise correlations between the rows of *H*. Entry *C*_*ij*_ in *C* represents the Spearman correlationbetween metagene *i* and metagene *j*. A cut-off threshold of *α* = 10^-12^ was set, and a connection was drawn between two nodes *i* and *j* if the p-value of the Spearman correlation between metagenes *i* and *j* was less than *α*.

The Spearman p-value was approximated using a two-sided Student's t-test. Specifically, if *r* is the Spearman correlation, and given that *n* = 783, the test-statistic *t* is given by:

(3) $$t=r\sqrt{\frac{n-2}{1-r^{2}}}$$

This statistic approximately follows a Student-t distribution with 781 degrees of freedom under the null hypothesis.

The pie chart representation of the network nodes, showing enrichment in gene ontologies, was created using the software library *ggplot2*[29] in R [25]. The metagenes were enriched in more ontologies than it was possible to display in the network graphs, so many of the ontologies were collapsed into their higher-level parents. Complete information on enrichment in all of the ontologies can be found in Additional files 2,3, and 4. Correlations between nodes were computed using the *corr* function in Matlab. The network graph was created in Cytoscape [30].

### Metagene Involvement in Experimental Series

The rows of *H* in (1) are the encoding coefficients for the *k* metagenes of *W*. Entry *h*_*ij*_ in *H* represents the activity level of metagene *i* in sample *j* of the dataset. While the columns of *W* provide us with functionally correlated groups of genes, each row of *H* represents the activation levels of one metagene across all samples. There are 783 samples in our dataset, divided into 29 different experimental series (see Table 1).

Through a convenient application of the Kruskal-Wallis test we can determine on which series of experiments each metagene is active on. The Kruskal-Wallis test is a non-parametric method for testing the equality of different population medians between groups [18]. The groups in our study are the 29 different experimental series in Table 1. The test is identical to a one-way analysis of variance test, except that the data values are replaced by ranks. It does not assume the data is drawn from a normal distribution.

Data measurements are converted to ranks before the test is applied. Given one row of *H*, the lowest activation level would be assigned a rank of 1, and the highest a rank of 783.

Given a row of *H* corresponding to one of the *k* metagenes, the *z*-value for group *j*, *j* = 1...29, is given by:

(4) $$z_{j}=\frac{{\bar{R}}_{j}-\bar{R}}{\sqrt{\frac{\left(N+1\right)\left(\frac{N}{N_{j}}-1\right)}{12}}}$$

where:

(6) $$\bar{R_{j}}=\left\{\text{mean}\;\text{rank}\;\text{of}\;\text{group}\;j\right\}$$

(7) $$\bar{R}=\left\{\text{mean}\;\text{of}\;\text{all}\;\text{ranks}\right\}$$

(8) $$N=\left\{\text{Total}\;\text{number}\;\text{of}\;\text{samples}\right\}$$

(9) $$N_{j}=\left\{\text{Number}\;\text{of}\;\text{samples}\;\text{in}\;\text{group}\;j\right\}\text{.}$$

The *z*-values are used to measure the activity level of each metagene on each of the 29 experimental series. They indicate the difference in the mean rank of the group from the mean rank of all observations. A positive *z*-value means that the metagene is active on that experiment, and a negative value indicates that it is suppressed. Storing the *z*-values representing metagene activity on each of the 29 different experimental series resulted in a 15×29 matrix. The result is displayed as a heat map (see Figure 2), and the spreadsheet is available as Additional file 6. Calculations for the Kruskal-Wallis test were carried out in Minitab.

### Gene Set Enrichment Analysis

The GSEA software package available from The Broad Institute (http://www.broadinstitute.org/gsea/index.jsp) [21,31], was used to functionally annotate the metagenes returned by NMF.

The GSEA algorithm takes as inputs two sets of gene lists:

1. A pre-defined set of genes S (such as genes sharing the same metabolic pathways)

2. A list of genes L ordered according to some ranking criteria of importance to the researcher.

In this study, we used the columns of *W* in the NMF factorization V≈WH to rank the genes. Entry *w*_*ij*_ of *W* represents the rank of gene *i* in metagene *j*. Since the metagenes represent local groups of highly correlated genes, it was felt that this was the best way to rank them.

Three databases of *Arabidopsis* gene sets involved in the gene ontologies of biological processes, cellular components, and molecular functions were used to functionally annotate the metagenes.

## Competing Interests

The authors declare that they have no competing interests.

## Authors’ contributions

TJW and SXG conceived the project. TJW did the analysis and wrote the paper. YB and LL created the GSEA knowledge base used in the enrichment analysis. All authors have read and approve the final manuscript.

## Supplementary Material

Additional file 1 **metagenes.** Excel spreadsheet containing the gene lists for each metagene as well as the gene ranks returned by NMF. Click here for file

Additional file 2 **GOBP.** Excel spreadsheet containing GSEA Enrichment results for Biological Processes. Click here for file

Additional file 3 **GOCC.** Excel spreadsheet containing GSEA Enrichment results for Cellular Components. Click here for file

Additional file 4 **GOMF.** Excel spreadsheet containing GSEA Enrichment results for Molecular Functions. Click here for file

Additional file 5 **Figure S1.** A plot showing the average correlation for 1000 randomly selected genes before and after the Empirical Bayes method was applied to adjust for batch effects. **S2**. A plot showing heat maps of the consensus matrix for k = {5,10,15,20}. **S3**. A plot showing heat maps of the consensus matrix for k = {30,35,40,45}. **S4.** A histogram of metagene coefficients, showing the δ=0.2 cut-off. Genes with coefficients greater than δ were included in the metagenes, and those with coefficients less than this were excluded. **S5**. Metagene correlation network for the gene ontology: cellular components. Each node in this network represents a metagene. The size of each node is proportional to the activity of the metagene within the dataset. The width of lines between a pair of nodes is proportional to the strength of the correlation between them. Positive correlations are denoted by red lines, and negative correlations by green lines. Only Spearman correlations with a p-value less than 10^-12^ are visible. The pie slices within each node represent the amount of enrichment for specific gene ontologies (the NES score). **S6**. Metagene correlation network for the gene ontology: molecular functions. Each node in this network represents a metagene. The size of each node is proportional to the activity of the metagene within the dataset. The width of lines between a pair of nodes is proportional to the strength of the correlation between them. Positive correlations are denoted by red lines, and negative correlations by green lines. Only Spearman correlations with a p-value less than 10^-12^ are visible. The pie slices within each node represent the amount of enrichment for specific gene ontologies (the NES score). **S7**. This heat map shows the z-values for all metagenes for the pathogen series in the dataset. Red indicates a metagene is more active in an experimental series, and green indicates it is suppressed. **S8**. Intersection p-values between clusters in the pathogen network from Atias, and the metagenes active in the pathogen series of the AtGenExpress dataset. Bright green cells represent significant statistical overlap. The intensity of the cells represents a log-10-transformed p-value returned by the hypergeometric test. **S9**. The NES score plotted in this heat map is a measure of metagene enrichment within a specific gene ontology involved with cellular components. Bright red cells indicate high enrichment. **S10**. The NES score plotted in this heat map is a measure of metagene enrichment within a specific gene ontology involved with molecular functions. Bright red cells indicate high enrichment. Click here for file

Additional file 6 **kruskal_wallis.** Excel spreadsheet containing z-values returned by the Kruskal-Wallis test on the encoding coefficients. Click here for file
