# Supplementary material for: Identification of metagenes and their Interactions through Large-scale Analysis of Arabidopsis Gene Expression Data
Source: BMC Genomics. 2012 Jun 13;13:237. doi: 10.1186/1471-2164-13-237 (PMC3536586; doi:10.1186/1471-2164-13-237)
Supplement: Additional file 5 — Figure S1. A plot showing the average correlation for 1000 randomly selected genes before and after the Empirical Bayes method was applied to adjust for batch effects. S2. A plot showing heat maps of the consensus matrix for k = {5,10,15,20}. S3. A plot showing heat maps of the consensus matrix for k = {30,35,40,45}. S4. A histogram of metagene coefficients, showing the δ=0.2 cut-off. Genes with coefficients greater than δ were included in the metagenes, and those with coefficients less than this were excluded. S5. Metagene correlation network for the gene ontology: cellular components. Each node in this network represents a metagene. The size of each node is proportional to the activity of the metagene within the dataset. The width of lines between a pair of nodes is proportional to the strength of the correlation between them. Positive correlations are denoted by red lines, and negative correlations by green lines. Only Spearman correlations with a p-value less than 10-12 are visible. The pie slices within each node represent the amount of enrichment for specific gene ontologies (the NES score). S6. Metagene correlation network for the gene ontology: molecular functions. Each node in this network represents a metagene. The size of each node is proportional to the activity of the metagene within the dataset. The width of lines between a pair of nodes is proportional to the strength of the correlation between them. Positive correlations are denoted by red lines, and negative correlations by green lines. Only Spearman correlations with a p-value less than 10-12 are visible. The pie slices within each node represent the amount of enrichment for specific gene ontologies (the NES score). S7. This heat map shows the z-values for all metagenes for the pathogen series in the dataset. Red indicates a metagene is more active in an experimental series, and green indicates it is suppressed. S8. Intersection p-values between clusters in the pathogen network from Atias, and the [file 1471-2164-13-237-S5.docx]

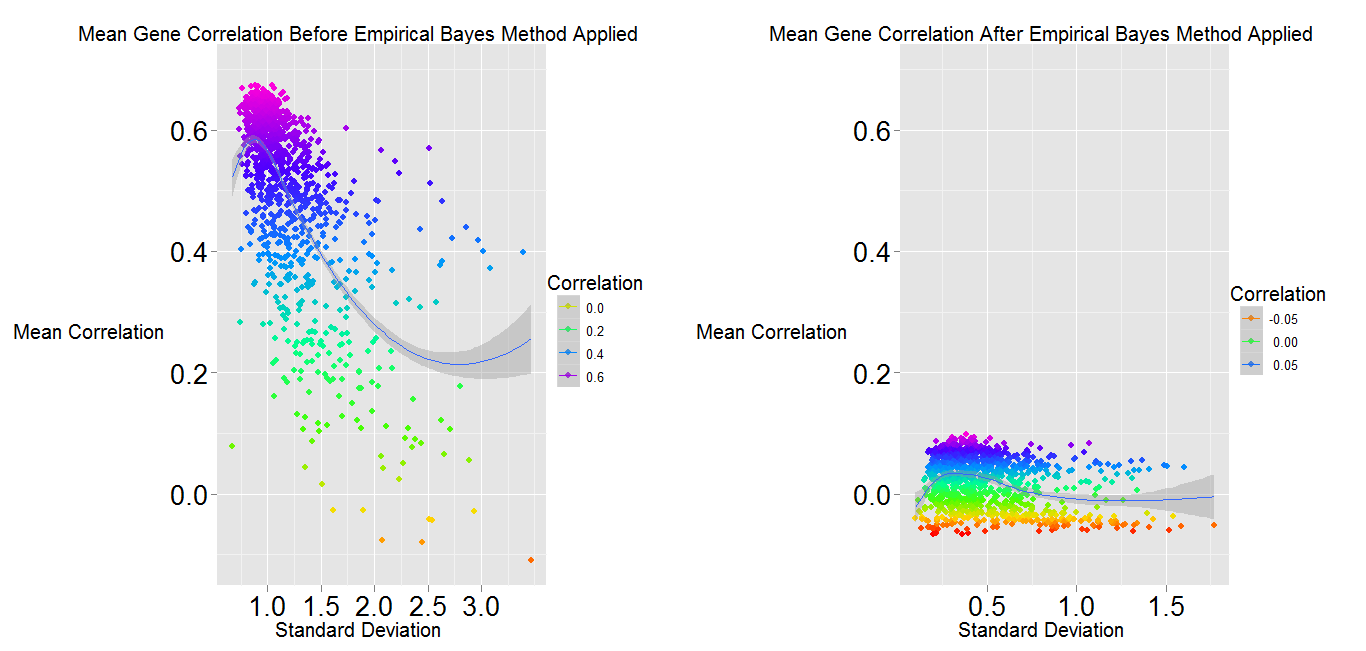


Figure S1: A plot showing the average correlation for 1000 randomly selected genes before and after the Empirical Bayes method was applied to adjust for batch effects.


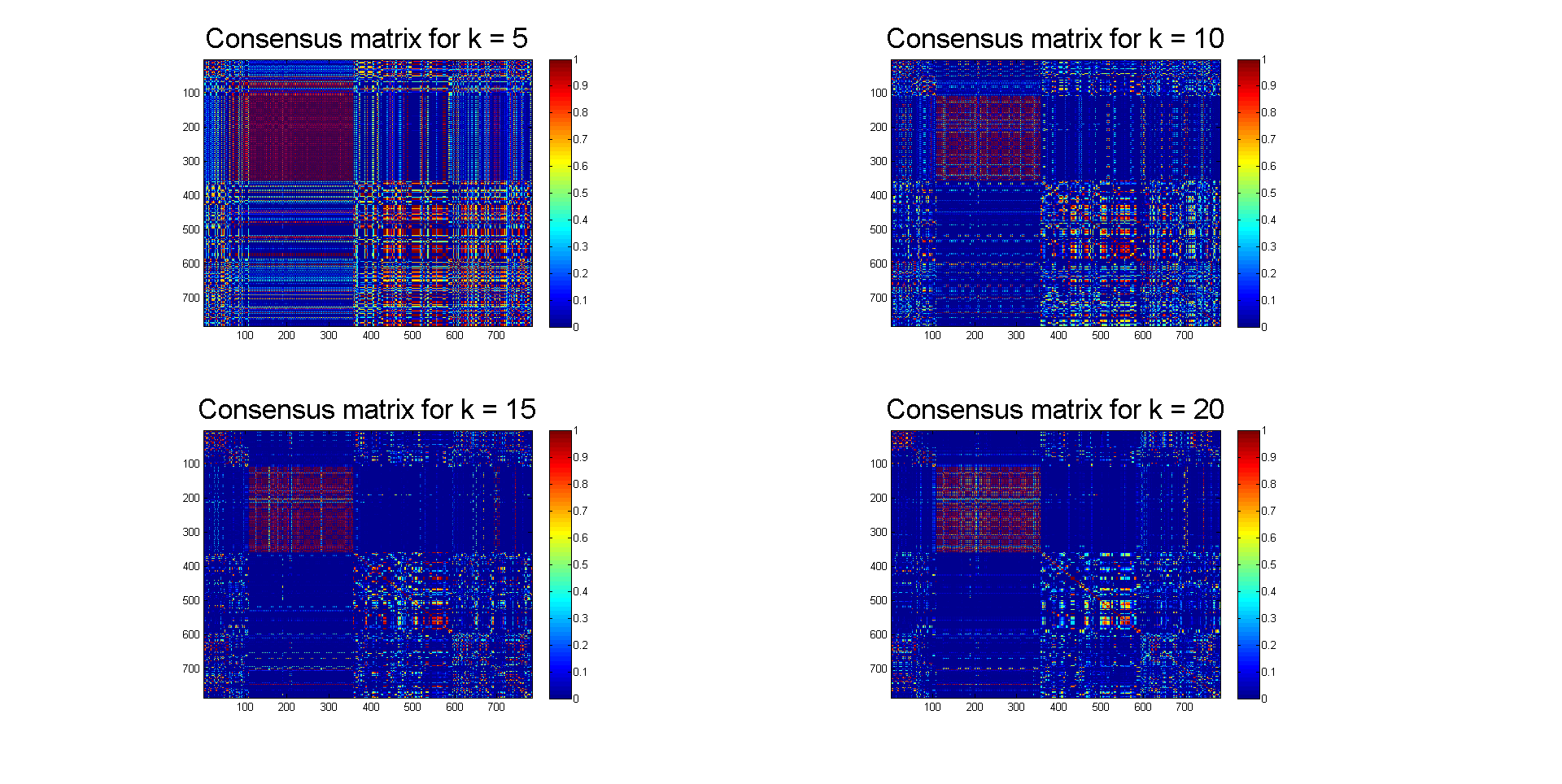


Figure S2: A plot showing heat maps of the consensus matrix for k = {5,10,15,20}


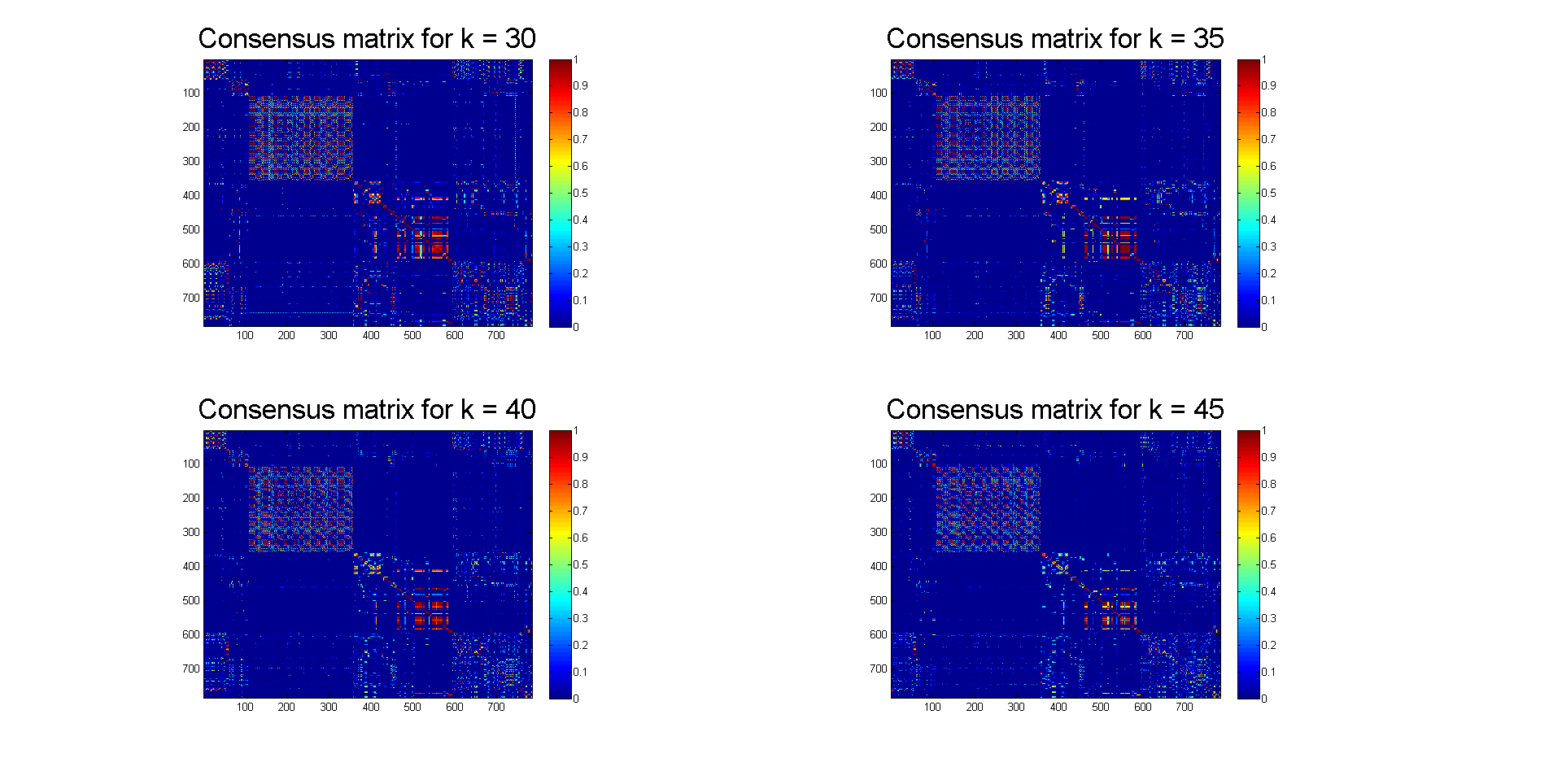


Figure S3: A plot showing heat maps of the consensus matrix for k = {30,35,40,45}


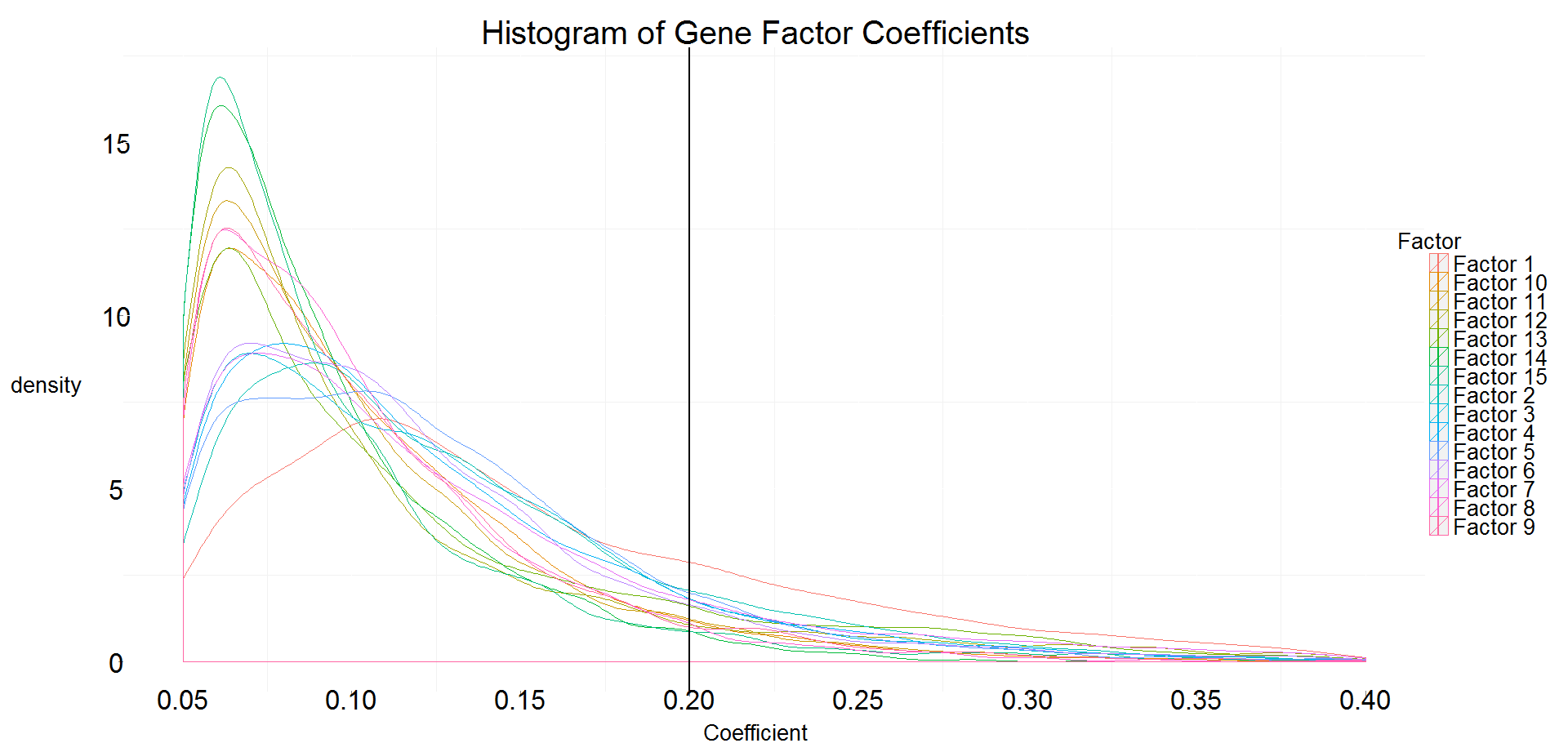


Figure S4: A histogram of metagene coefficients, showing the δ=0.2 cut-off. Genes with coefficients greater than δ were included in the metagenes, and those with coefficients less than this were excluded.


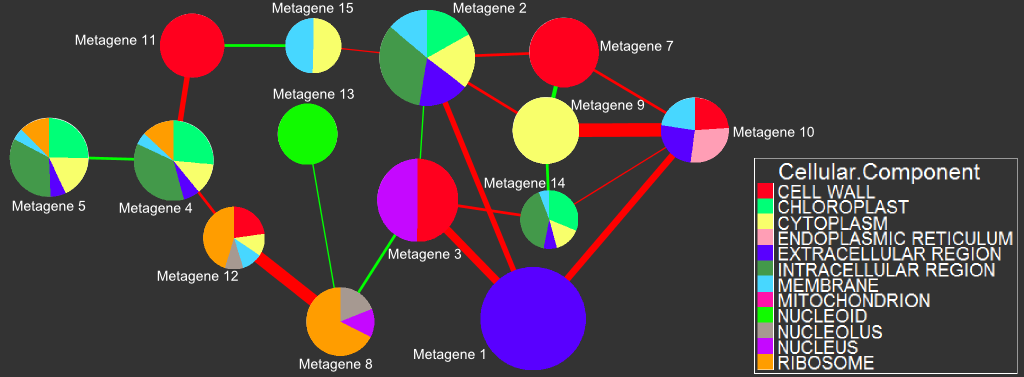


Figure S5: Metagene correlation network for the gene ontology: cellular components. Each node in this network represents a metagene. The size of each node is proportional to the activity of the metagene within the dataset. The width of lines between a pair of nodes is proportional to the strength of the correlation between them. Positive correlations are denoted by red lines, and negative correlations by green lines. Only Spearman correlations with a p-value less than 10^-12^ are visible. The pie slices within each node represent the amount of enrichment for specific gene ontologies (the NES score).


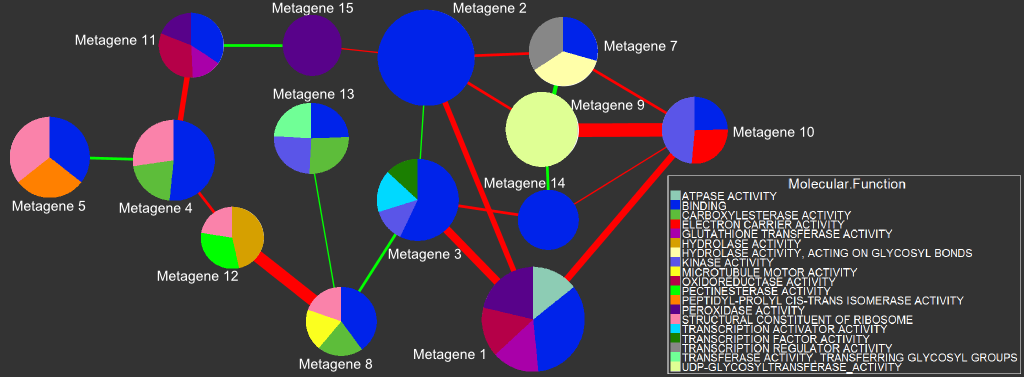


Figure S6: Metagene correlation network for the gene ontology: molecular functions. Each node in this network represents a metagene. The size of each node is proportional to the activity of the metagene within the dataset. The width of lines between a pair of nodes is proportional to the strength of the correlation between them. Positive correlations are denoted by red lines, and negative correlations by green lines. Only Spearman correlations with a p-value less than 10^-12^ are visible. The pie slices within each node represent the amount of enrichment for specific gene ontologies (the NES score).


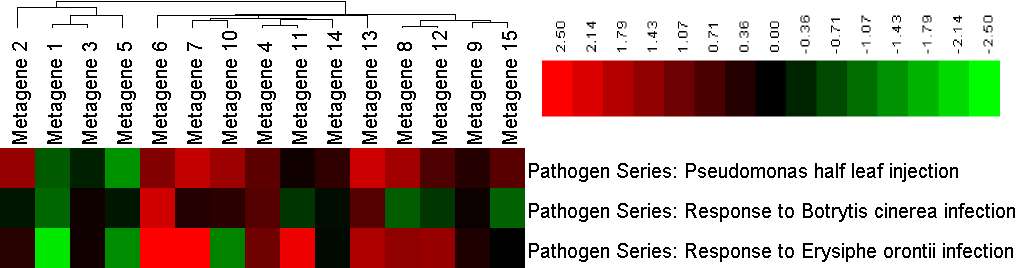


Figure S7: This heat map shows the z-values for all metagenes for the pathogen series in the dataset. Red indicates a metagene is more active in an experimental series, and green indicates it is suppressed.


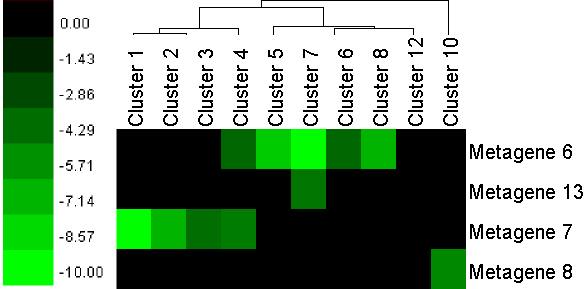


Figure S8: Intersection p-values between clusters in the pathogen network from Atias, and the metagenes active in the pathogen series of the AtGenExpress dataset. Bright green cells represent significant statistical overlap. The intensity of the cells represents a log-10-transformed p-value returned by the hypergeometric test.


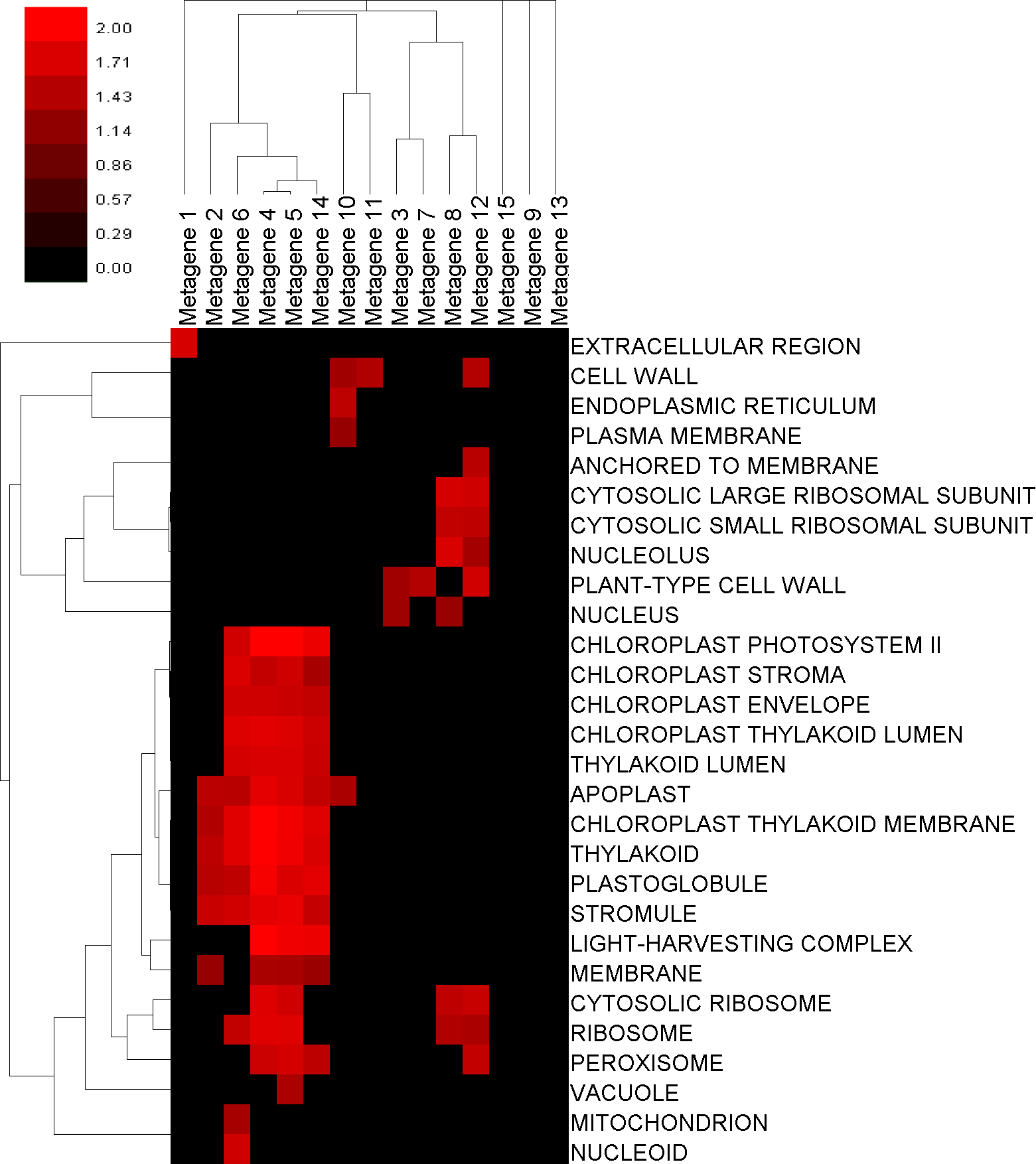


Figure S9: The NES score plotted in this heat map is a measure of metagene enrichment within a specific gene ontology involved with cellular components. Bright red cells indicate high enrichment.


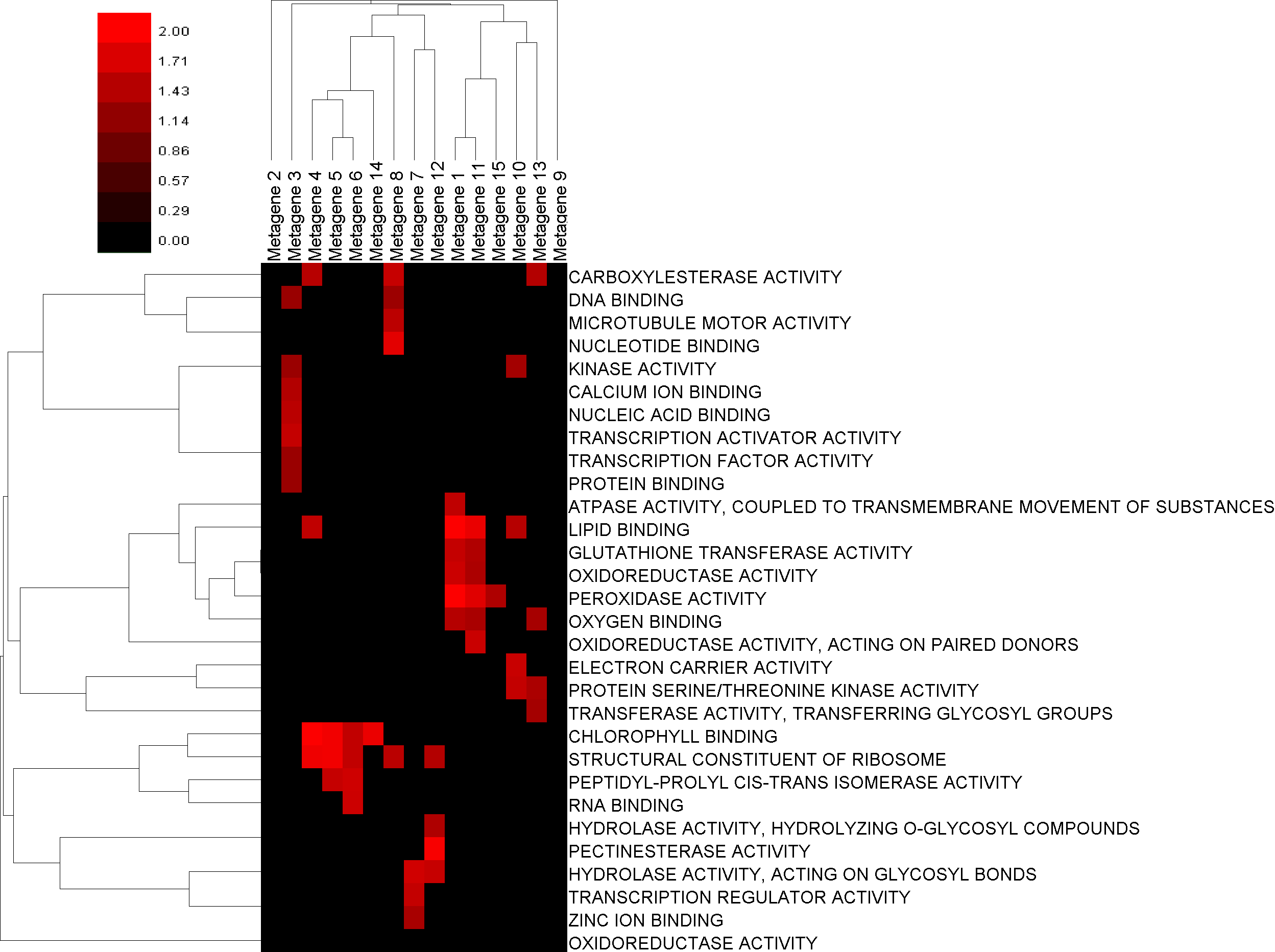


Figure S10: The NES score plotted in this heat map is a measure of metagene enrichment within a specific gene ontology involved with molecular functions. Bright red cells indicate high enrichment.


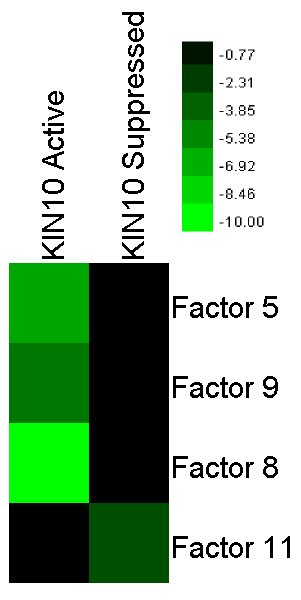


Figure S11: Intersection p-values between KIN10 target gene groups, and the metagenes in this study. Bright green cells represent significant intersections. The intensity of the cells represents a log10-transformed p-value returned by the hypergeometric test.
